# Pyruvate dehydrogenase alleviates macrophage autophagy in Hcy-induced ApoE ^–/–^ mice: Homocysteine suppresses macrophage autophagy by inhibiting PDH

**DOI:** 10.3724/abbs.2025021

**Published:** 2025-02-20

**Authors:** Qiujun Liu, Feng Li, Shutong Hu, Ning Ding, Fang Ma, Yinju Hao, Guizhong Li, Jiantuan Xiong, Huiping Zhang, Yideng Jiang

**Affiliations:** 1 NHC Key Laboratory of Metabolic Cardiovascular Diseases Research Ningxia Medical University Yinchuan 750004 China; 2 Department of Medical Genetics Maternal and Child Health of Hunan Province Changsha 410008 China; 3 Ningxia Key Laboratory of Vascular Injury and Repair Research Ningxia Medical University Yinchuan 750004 China; 4 School of Basic Medical Sciences Ningxia Medical University Yinchuan 750004 China; 5 Center of Laboratory Medicine General Hospital of Ningxia Medical University Yinchuan 750004 China; 6 Medical Experimental Center General Hospital of Ningxia Medical University Yinchuan 750004 China

**Keywords:** pyruvate dehydrogenase activity, homocysteine, atherosclerosis, autophagy

## Abstract

Macrophages play a protective role in atherosclerosis, whereas homocysteine (Hcy) is recognized as an independent risk factor for atherosclerosis. Defects in macrophage autophagy contribute to the formation of atherosclerotic plaques, and dysregulated energy metabolism is closely linked to the process of autophagy. However, the regulation of macrophage autophagy by pyruvate dehydrogenase (PDH), a key component of the PDH complex involved in energy and metabolic homeostasis, remains poorly understood in the context of atherosclerosis induced by Hcy. In our study, proteomic profiling identifies 748 upregulated proteins and 760 downregulated proteins in Hcy-treated macrophages. KEGG pathway analysis reveals significant enrichment of differentially expressed proteins in metabolism-related pathways, including those related to the biosynthesis of amino acids, carbon metabolism, and glycolysis/gluconeogenesis. Additionally, we explore the role of PDH in mediating Hcy-induced atherosclerosis in ApoE ^– **/**–^ mice. The results show a marked reduction in PDH expression and activity in Hcy-treated macrophages, leading to impaired autophagy. Notably, PDH activation enhances the assembly of the autophagy initiator ULK1-FIP200-Atg13 complex through the modulation of the AMPK/mTOR signaling pathway, suggesting a potential therapeutic target for Hcy-induced atherosclerosis.

## Introduction

Atherosclerosis is a major cause of many cardiovascular diseases and is strongly associated with global mortality and morbidity [ 1, 2]. Numerous studies have identified homocysteine (Hcy) as an independent risk factor for atherosclerosis, as it induces endothelial dysfunction, abnormal proliferation of vascular smooth muscle cells, and other pathological changes [ 3, 4]. Previous research has shown that inflammation increases in macrophages as plaque progresses, highlighting the critical role of macrophages in the development of atherosclerotic lesions [5]. However, the underlying mechanisms by which macrophages contribute to Hcy-induced atherosclerosis remain unclear.

Autophagy is an evolutionarily conserved process that sequesters excess, aged, or damaged cytoplasmic material and delivers it to lysosomes for degradation. Efficient autophagy clears damaged organelles and proteins resulting from cellular stress and damage. It also degrades cholesterol ingested by macrophages before it is effluxed, thereby preventing lipid accumulation and foam cell formation in atherosclerosis [ 6, 7]. However, defects in autophagy have been associated with an increased risk of specific cardiovascular disorders in laboratory animals [ 8, 9]. For example, dysregulated autophagy can contribute to ischaemia‒reperfusion injury and postmyocardial infarction [ 10– 12]. Moreover, Yang *et al*. [13] demonstrated that Hcy could accelerate hepatocyte autophagy by upregulating TFEB through DNMT3b-mediated DNA hypomethylation. Autophagy is regulated by the AMPK/mTOR/ULK1 signaling pathway [ 14, 15], and ULK1 is activated by serine-threonine protein kinases and forms a complex with ATG13 and FIP200, initiating autophagy [16]. These findings underscore the need for further research to elucidate the mechanisms of autophagy in Hcy-induced atherosclerosis.

Numerous studies have demonstrated that glycolysis and the mitochondrial tricarboxylic acid (TCA) cycle are involved in various pathological processes of cardiovascular diseases, including endothelial dysfunction, inflammation, vascular smooth muscle cell proliferation, and thrombosis following plaque rupture [ 17, 18]. Pyruvate dehydrogenase (PDH), a key rate-limiting enzyme that catalyzes irreversible oxidative decarboxylation of pyruvate, links glycolysis with the TCA cycle and oxidative phosphorylation and regulates its activity to ensure the balance of cellular energy metabolism [19]. Recent studies have highlighted the relationship between PDH activity and several malignancies, such as colorectal cancer and renal cancer [ 20, 21]. In addition to cancer, PDH dysfunction has been implicated in cardiovascular diseases, including hypertension, myocarditis, and type 2 diabetes [ 22, 23]. Additionally, Sojeong *et al*. [24] reported that PDH deficiency resulted in a reduction in the number of double-positive T-cell progenitor cells, which contributed to leukemia development. Despite these insights, the mechanisms and role of the PDH in atherosclerosis remain poorly understood.

In the present study, we demonstrated that Hcy inhibits macrophage autophagy by suppressing PDH expression and activity. Furthermore, the activation of PDH reversed the suppression of autophagy in Hcy-treated macrophages. Our findings also revealed that a PDH activator alleviates autophagy through the AMPK/mTOR signaling pathway in Hcy-treated macrophages. These results provide compelling evidence for the potential therapeutic efficacy of targeting PDH to inhibit atherosclerosis development.

## Materials and Methods

### Animals

All experimental procedures and animal care adhered strictly to the detailed rules and regulations set forth by the Ministry of Public Health of China for Medical Animal Experimentation Administration and Implementation. Apolipoprotein-E knockout (ApoE ^–/–^) mice with a male C57BL/6J background aged six weeks were procured from Weishang Lide Biotechnology (Beijing, China). After a two-week adaptation period for the atherosclerotic models, 8-week-old mice were fed a normal chow diet (CD: 20% protein, 4.5% fat, 55.5% carbohydrate, *n* = 6) or a 1.7% high-methionine diet (HMD, *n* = 6) for 22 weeks. To assess the therapeutic effect of the PDH activator DCA (dissolved in PBS, *n* = 6) on the progression of atherosclerosis, DCA (1 mg/kg) was intraperitoneally injected every other day for 4 weeks following 26 weeks of HMD. The control mice received intraperitoneal injections of vehicle (PBS). After being fed their respective diets for a total duration of 30 weeks, the mice were euthanized with pentobarbital (50 mg/kg body weight). Aortic tissues were collected and frozen in liquid nitrogen at –80°C until further analysis.

### Cell culture

Mouse macrophages (RAW264.7) were obtained from the Cell Bank of the Chinese Academy of Sciences (Shanghai, China), cultured in high-DMEM (Gibco, Carlsbad, USA) supplemented with 10% fetal bovine serum (FBS; Gibco), and maintained at 37°C in a humidified atmosphere containing 5% CO _2_. The macrophages were subsequently divided into different groups: the control group was supplemented with 7% high-DMEM without Hcy for 24 h, the Hcy group was exposed to 100 μM Hcy for 24 h, the 2-DG group was subjected to treatment with 10 μM 2-deoxy-D-glucose (2-DG, HY-13966; MCE, New Jersey, USA), the CPI-613 group was treated with 1.25 μM devimistat (CPI-613, HY-15453; MCE), and the bafilomycin A1 (BafA1, HY-100558; MCE) group was treated with 2.5 μM BafA1 for 24 h.

### Proteomic profiling

Macrophages were lysed in 8 M urea buffer containing 1 mM PMSF and 2 mM EDTA, followed by 5 min of on-ice ultrasonication. After centrifugation at 15,000 *g* for 10 min at 4°C, the supernatant was collected. Proteins were reduced with 10 mM DTT at 37°C for 45 min and alkylated with 50 mM IAM for 15 min in the dark. The proteins were precipitated with cold acetone at –20°C for 2 h, then digested overnight with trypsin in 25 mM ammonium bicarbonate at 37°C. The peptides were desalted on a C18 column, concentrated, and redissolved in 0.1% formic acid for HPLC-MS/MS analysis. HPLC-MS/MS separate and identify peptides. By detecting the mass and fragmentation information of peptides, it is possible to determine the amino acid sequence and modification status of proteins, thus enabling the qualitative and quantitative analysis of proteins in macrophages.

### RNA interference and transfection

The oligonucleotide sequences used for RNA interference (RNAi) were as follows: negative control 5′-UUCUCCGAACGUGUCACGUTT-3′ (sense) and 5′-ACGUGACACGUUCGGAGAATT-3′ (antisense); PDH siRNA 5′-GGAAUUGAAUGUGAGGUAATT-3′ (sense) and UUACCUCACAUUCAAUUCCTT-3′ (antisense). All the RNAi oligonucleotides were obtained from GeneCarer Company (Xi’an, China). For siRNA transfection, a mixture of 5 μL of siRNA and 5 μL of INVI DNA RNA Transfection Reagent (Invitrogen, Carlsbad, USA) was incubated at room temperature for 15 min; then, the mixture was added to 2 mL of the medium supernatant of the macrophages. After incubation for 48 h, total protein was extracted from the macrophages for western blot analysis.

### Measurement of pyruvate dehydrogenase (PDH) activity

PDH, a rate-regulating enzyme catalyzed by the pyruvate dehydrogenase complex (PDHc), catalyzes the oxidative decarboxylation of pyruvate to form hydroxyethyl-TPP, which links glycolysis and the TCA cycle. PDH catalyzes pyruvate dehydrogenation and reduces 2,6-dichlorophenol indophenol (2,6-dCPIP), resulting in a reduction in light absorption at 605 nm. A total of 5 × 10 ^7^ macrophages were collected in a centrifuge tube, and the supernatant was discarded. After adding 1 mL of reagent 1 and 10 μL of reagent 2, the macrophages were disrupted via an ice bath ultrasonic wave (power of 200 W, ultrasonication for 3 s, interval of 7 s, and total time of 5 min). The samples were centrifuged at 11,000 *g* and 4°C for 10 min, and the supernatant was collected and placed on ice for measurement. The absorbance value was subsequently detected by an enzyme at 605 nm, and the PDH activity was subsequently calculated according to the amount of cell protein (BC0385; Solarbio, Beijing, China).

### Measurement of pyruvate (PA) content

A total of 5 × 10 ^7^ macrophages were collected in a centrifuge tube, 1 mL of extraction solution was added, ultrasonic crushing was performed (ice bath, power 200 W, ultrasonication 3 s, 10 s interval, repeated 30 times), the macrophages were left for 30 min at 8000 *g*, the mixture was centrifuged at room temperature for 10 min, and the supernatant was collected for measurement. Then, the absorbance value was detected at 520 nm by an enzyme, and the PA content was calculated according to the amount of cell protein (Solarbio).

### Measurement of the glucose content

A total of 5 × 10 ^7^ macrophages were added to 1 mL of distilled water, after which the macrophages were disrupted via ultrasound (ice bath, 200 W, ultrasonication for 3 s, 10 s intervals, 30 cycles), boiled in a boiling water bath for 10 min (cover tightly to prevent water loss), cooled at 8000 *g*, and centrifuged at 25°C for 10 min, after which the supernatant was removed for use. The absorbance value was subsequently detected at 505 nm by an enzyme, and the glucose content was subsequently calculated according to the amount of cell protein (Solarbio).

### Co-immunoprecipitation (Co-IP) assays

Cells were lysed in cold 1 mL NP-40 lysis buffer (G-CLONE, Beijing, China) supplemented with 10 μL PMSF (Solarbio) for 2 h, and Lysates were centrifuged at 12,000 *g* for 2 h at 4°C, and supernatants were quantified using a BCA assay (Keygen Biotech, Nanjing, China). Then, pre-cleared lysates were incubated at 4°C with 4 μg of ULK1 antibody (1:100, 8054; Cell Signaling Technology, Danvers, USA) and IgG isotype control (1:100, AC005; ABclonal, Wuhan, China) for 2 h. A total of 30 μL Pierce ^*^ Protein A/G Agarose beads (88803; Thermo Fisher, Waltham, USA) were then added, followed by incubation for overnight. Beads were washed 3 times with lysis buffer, and bound proteins were eluted by boiling in 2× Proteintechloading buffer (CW0027S; CWBIO, Beijing, China) at 99 ^°^C for 10 min. Eluted proteins were analysis via western blot analysis.

### Western blot analysis

The macrophages were extracted with lysis buffer (Keygen Biotech). The lysates were subsequently centrifuged, and the supernatants were collected. Protein samples were resolved by SDS-PAGE and transferred to polyvinylidene fluoride membranes (Millipore, Billerica, USA) using a semidry transfer system (Bio-Rad, Hercules, USA). Following this step, the membranes were blocked with 5% BSA at room temperature for 2 h and subsequently immunoblotted overnight with the indicated antibodies against PDH (1:1000, ET1705-57; HUABIO, Hangzhou, China), LC3B (1:1000, ab192890; Abcam, Cambridge, UK), p62 (1:1000, ab109012; Abcam), p-AMPK (T172) (1:1000, ab314032; Abcam), AMPK (1:1000, ab32047; Abcam), p-ULK (S317) (1:1000, 89267; Cell Signaling Technology), ULK (1:1000, 8054; Cell Signaling Technology), p-mTOR (S2448) (1:1000, 5536; Cell Signaling Technology), mTOR (1:1000, 2983; Cell Signaling Technology), Atg13 antibody (1:1000, TN22590; Abmart, Shanghai, China), and FIP200 (1:1000, MU601106; Abmart). This was followed by incubation with a horseradish peroxidase-conjugated Goat Anti-Rabbit IgG (HRP) (1:2500, AB0101; Abways, Shanghai, China). Specific protein bands were visualized by enhanced chemiluminescence (Beyotime, Shanghai, China) and quantified via densitometry analysis via Image Lab software.

### Analysis of autophagic flux

The assessment of autophagic flux was conducted using mRFP-GFP-LC3 adenovirus vectors (Hanheng, Hangzhou, China). Macrophages were transfected with the vectors via INVI DNA RNA Transfection Reagent (Invitrogen), followed by visualization of GFP and mRFP expression via laser scanning confocal microscopy (Olympus, Tokyo, Japan). The autophagosomes were identified as yellow puncta (mRFP ^+^ and GFP ^–^), whereas the autolysosomes were identified as red puncta (mRFP ^+^ and GFP ^–^). The relative ratio of red/yellow puncta served as an indicator for quantifying autophagic flux. In summary, mRFP-GFP-LC3 adenovirus vectors were employed to monitor autophagic flux on the basis of the expression levels of GFP and mRFP. The ratio of red to yellow puncta was used to quantify the extent of autophagic flux.

### Immunofluorescence

Frozen sections of aortic roots were fixed in 4% paraformaldehyde for 15 min and permeabilized in 0.2% Triton X-100 for 15 min. Following blocking with 10% goat serum for 10 min, the sections were subsequently blocked with 5% goat serum at room temperature for 1 h. Primary antibodies against LC3B (1:200, ab192890; Abcam), p62 (1:200, ab109012; Abcam), or Mac-2 (1:200, ab316101; Abcam) were then added, and the samples were incubated overnight at 4°C. After being washed with PBS, the sections were incubated with FITC-conjugated secondary antibodies (1:200, sa00003-1; Proteintech, Wuhan, China) or Alexa Fluor 647 secondary antibodies (1:200, sms2bAF647; Proteintech) for 1 h at 37°C. The sections were subsequently washed with PBS, and the nuclei were stained with 4′,6-diamidino-2-phenylindole (DAPI) in the dark for 8 min. The fluorescence images were captured using a confocal microscope (Olympus).

### Pathway and functional enrichment analyses

To gain a comprehensive understanding of the biological functions of these genes and elucidate their potential mechanisms in macrophages, we employed the R clusterRrofiler package to conduct KEGG analyses. Gene set enrichment analysis (GSEA) was performed using GSEA software (version 4.1.0).

### Statistical analysis

The data analysis was performed using GraphPad Prism 8.0 software. Data in this study is presentedas the mean ± standard deviation (SD) from a minimum of three independent experiments. For the western blot and autophagic flux experiments, we carried out three independent experiments. Statistical differences between groups were evaluated by one-way analysis of variance combined with the Student-Newman-Keuls post hoc test or the nonparametric Mann-Whitney U test. A significance level of *P* < 0.05 was considered statistically significant.

## Results

### Hcy inhibits autophagy by regulating glycolysis in macrophages

**Figure 1:**
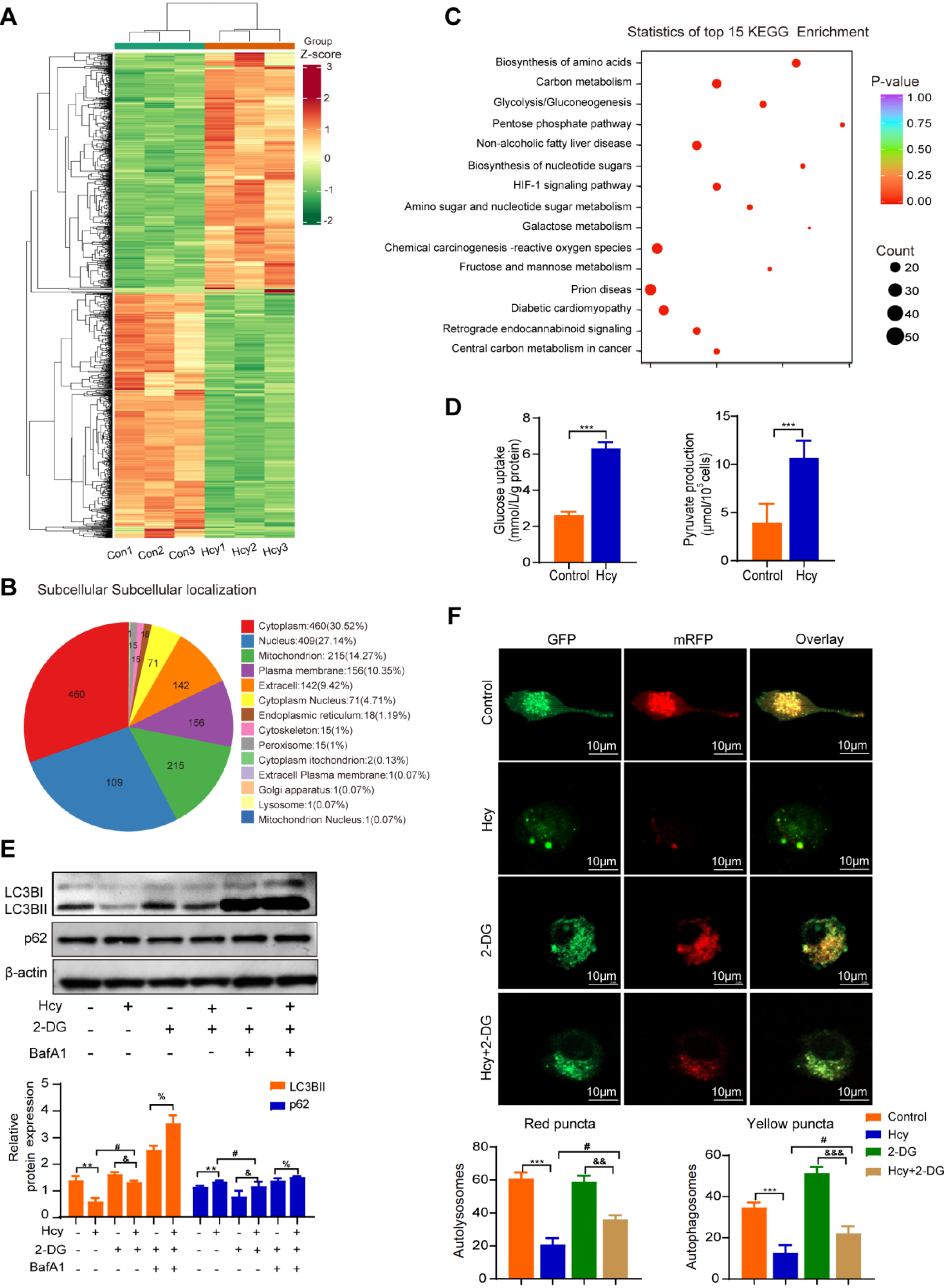
Figure 1 Hcy inhibits macrophage autophagy by regulating glycolysis (A) Quantification and characterization of differentially expressed proteins in macrophages treated with Hcy using high-performance liquid chromatography-tandem mass spectrometry (HPLC-MS/MS). (B) Pie chart showing the subcellular localization of the differentially modified proteins, with the numbers on each segment representing the protein number count. (C) KEGG analysis of the top 15 signaling pathways related to host genes producing differentially expressed proteins. (D) Glucose uptake and pyruvate production were detected in macrophages treated with or without Hcy. (E) The expression of LC3BII and p62 in macrophages treated with 2-DG or Bafa1 in the presence of Hcy was detected via western blot analysis. (F) Representative confocal fluorescence images of mRFP-GFP-LC3 in macrophages treated with 2-DG or Hcy. Yellow puncta and merged mRFP and GFP fluorescence indicate autophagosomes, whereas free red puncta (mRFP only) represent autolysosomes. The numbers of autophagosomes and autolysosomes in each cell were quantified. Scale bar: 10 μm. Data are presented as the mean ± SD. *P < 0.05, ***P < 0.001, control group vs Hcy group; &P < 0.05, 2-DG group vs Hcy + 2-DG group; #P < 0.05, Hcy group vs Hcy + 2-DG group; %P < 0.05, 2-DG+BafA1 group vs Hcy + 2-DG+BafA1 group.

To gain deeper insight into the potential mechanism of autophagy induction by Hcy in macrophages, we conducted proteomic profiling of macrophages treated with Hcy for 24 h. Following the criteria of a log2 |fold change| ≥ 1.2 and *P <*0.05, we identified 748 upregulated proteins and 760 downregulated proteins in Hcy-treated macrophages ( Figure 1A). These modified proteins are located in various organelles, predominantly in the cytoplasm and nucleus ( Figure 1B). To explore the functional relevance of these proteins, we conducted a KEGG pathway analysis, which revealed significant enrichment of differentially expressed proteins in pathways related to the biosynthesis of amino acids, carbon metabolism, and glycolysis/gluconeogenesis ( Figure 1C). Furthermore, we observed an increase in glucose uptake and pyruvate production in Hcy-treated macrophages, indicating that Hcy enhances glycolysis in these cells. ( Figure 1D). Given that Hcy has been shown to affect autophagy in hepatocytes [13], we examined its impact on macrophage autophagy. Western blot analysis revealed a decrease in LC3BII expression and an increase in p62 expression in Hcy-treated macrophages. However, treatment with 2-DG, a glycolysis inhibitor, counteracted this effect, leading to an increase in LC3BII expression and a decrease in p62 expression ( Figure 1E). Additionally, immunofluorescence analysis using mRFP-GFP-LC3 adenoviral vectors revealed marked accumulation of mRFP puncta and GFP puncta in the perinuclear region and cytoplasm of macrophages treated with 2-DG ( Figure 1F). Collectively, these results strongly suggest that Hcy suppresses autophagy by regulating glycolysis in macrophages.

### Hcy suppresses autophagy via the inhibition of pyruvate dehydrogenase in macrophages

**Figure 2:**
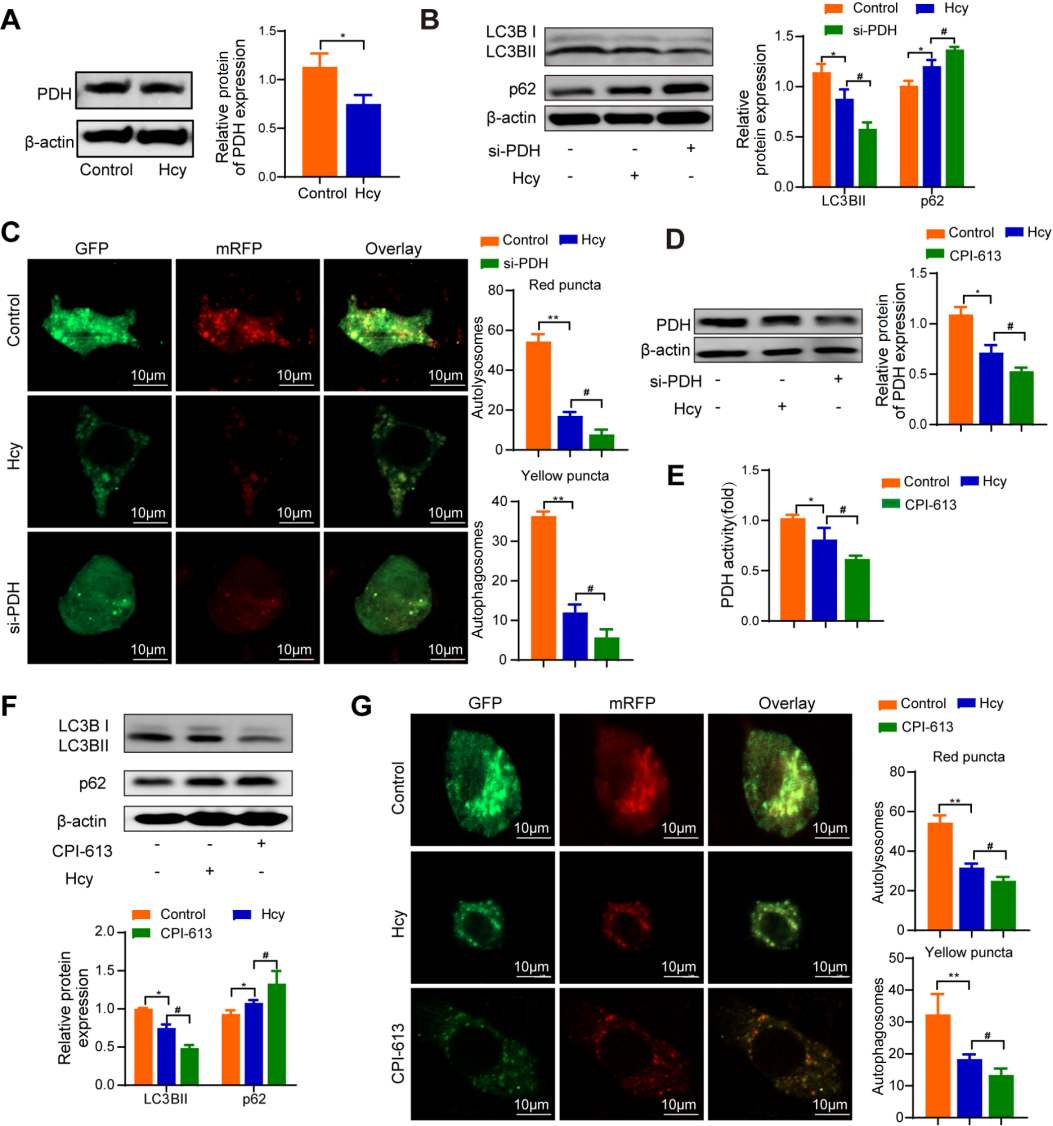
Figure 2 PDH is involved in macrophage autophagy induced by Hcy (A) PDH expression was detected by western blot analysis in macrophages treated without Hcy or with Hcy. (B) The expressions of LC3BII and p62 were detected by western blot analysis in macrophages transfected with si-PDH. (C) Representative confocal fluorescence images showing mRFP-GFP-LC3 in macrophages transfected with si-PDH. Yellow puncta, the merging of mRFP and GFP fluorescence, indicate autophagosomes, whereas free red puncta (mRFP only) represent autolysosomes. The numbers of autophagosomes and autolysosomes in each cell were quantified. (D) PDH expression was detected by western blot analysis in macrophages treated with Hcy or CPI-613. (E) PDH activity was detected in macrophages treated with Hcy or CPI-613. (F) The expressions of LC3BII and p62 in macrophages treated with Hcy or CPI-613 were detected by western blot analysis. (G) Representative confocal fluorescence images showing mRFP-GFP-LC3 in macrophages treated with Hcy or CPI-613. Yellow puncta, the merging of mRFP and GFP fluorescence, indicate autophagosomes, whereas free red puncta (mRFP only) represent autolysosomes. The numbers of autophagosomes and autolysosomes in each cell were quantified. Scale bar: 10 μm. Data are presented as the mean ± SD. *P < 0.05, **P < 0.01, control group vs Hcy group; #P < 0.05 Hcy group vs CPI-613 group .

PDH, a key regulator in mitochondria, plays an essential role in maintaining energy homeostasis and adapting to changes in metabolic conditions and energy demands [25]. Our study revealed that PDH expression was decreased in Hcy-treated macrophages ( Figure 2A). To investigate the relationship between PDH and macrophage autophagy, we conducted further experiments. Western blot analysis revealed that LC3BII expression was decreased, whereas p62 expression was increased in macrophages transfected with si-PDH ( Figure 2B). Additionally, mRFP-GFP-LC3 adenoviral vector analysis revealed a significant reduction in both mRFP puncta and GFP puncta in macrophages transfected with si-PDH ( Figure 2C). PDH is an important regulatory enzyme in the glycolysis pathway that serves as a key link between glycolysis and the TCA cycle and maintains the balance of cellular energy metabolism by regulating its activity. Therefore, we found that PDH activity was also decreased in macrophages treated with Hcy. Moreover, CPI-613, a potent PDH inhibitor, was able to inhibit both PDH expression and activity in macrophages ( Figure 2D,E). Furthermore, western blot analysis revealed that PDH expression was decreased in macrophages treated with CPI-613. To elucidate the role of CPI-613 in autophagy, western blot analysis revealed that LC3BII expression was decreased, whereas p62 expression was increased in macrophages treated with CPI-613 ( Figure 2F). Additionally, mRFP-GFP-LC3 adenoviral vector analysis demonstrated a significant reduction in mRFP puncta and GFP puncta in macrophages treated with CPI-613 ( Figure 2G). These findings indicate that Hcy suppresses macrophage autophagy by affecting pyruvate dehydrogenase.

### Activated PDH reverses Hcy-induced inhibition of macrophage autophagy

**Figure 3:**
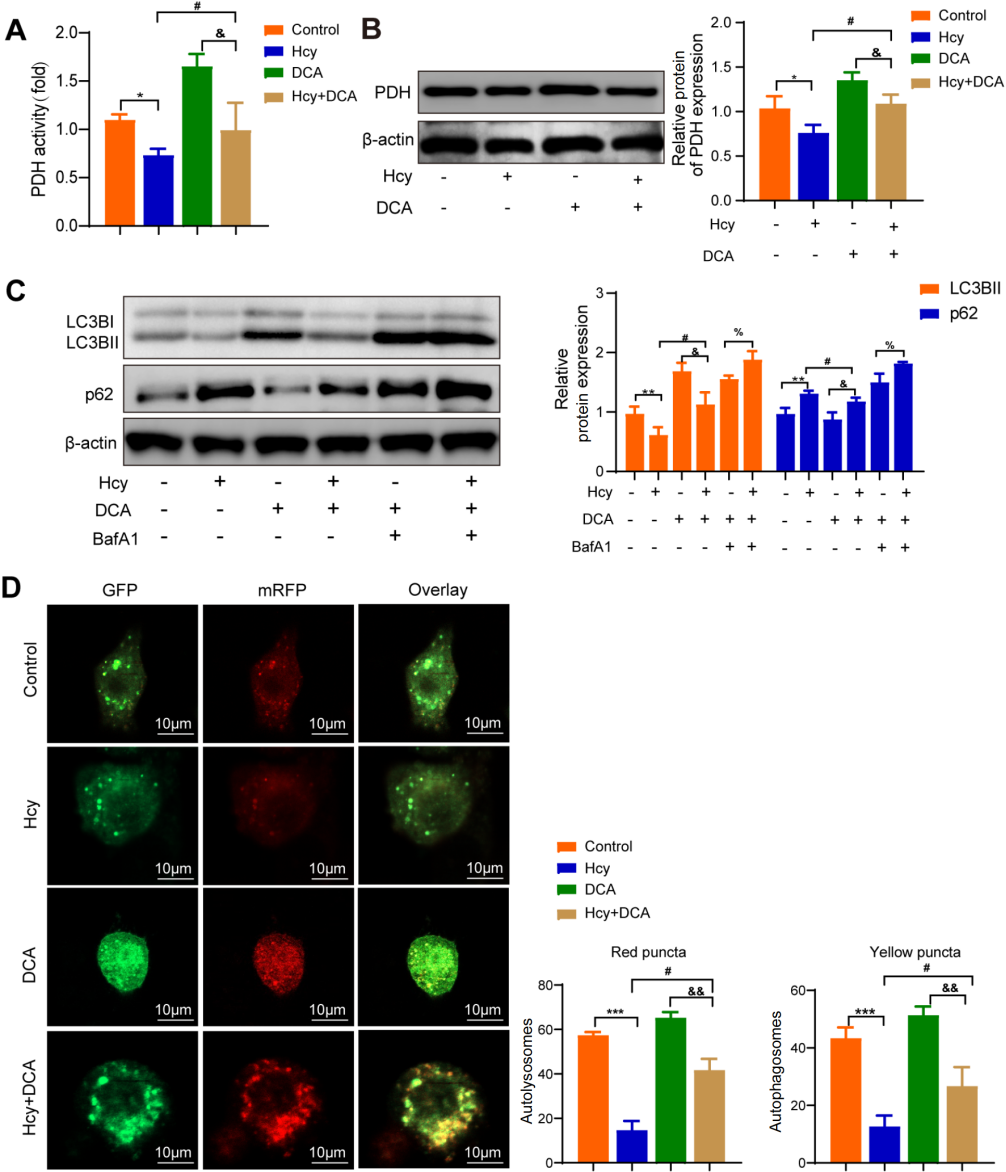
Figure 3 PDH activator facilitates autophagy in Hcy-induced macrophages (A) PDH activity was detected in macrophages treated with Hcy or DCA (dichloroacetate, a pyruvate dehydrogenase activator). (B) PDH expression was detected by western blot analysis in macrophages treated with Hcy or DCA. (C) The expressions of LC3BII and p62 in macrophages treated with DCA or BafA1 in the presence of Hcy were detected via western blot analysis. (D) Representative confocal fluorescence images of mRFP-GFP-LC3 in macrophages treated with DCA or Hcy. Yellow puncta and merged mRFP and GFP fluorescence indicate autophagosomes, whereas free red puncta (mRFP only) represent autolysosomes. The numbers of autophagosomes and autolysosomes in each cell were quantified. Scale bar: 10 μm. Data are presented as the mean ± SD. *P < 0.05, ***P < 0.001, control group vs Hcy group; &P < 0.05, DCA group vs Hcy + DCA group; #P < 0.05, Hcy group vs Hcy + DCA group; %P < 0.05, DCA + BafA1 group vs Hcy + DCA + BafA1 group.

DCA (a pyruvate dehydrogenase activator) inhibits pyruvate dehydrogenase kinase and reduces the phosphorylation of PDH, thereby activating pyruvate dehydrogenase [26]. To further investigate the effect of DCA on macrophage autophagy. As shown in Figure 3A,B, PDH activity and expression were decreased in macrophages treated with Hcy. However, the PDH activator DCA was able to counteract this effect. Western blot analysis revealed that LC3BII expression was increased, whereas p62 expression was decreased in macrophages treated with DCA. In contrast, LC3BII expression decreased, and p62 expression increased in macrophages treated with both Hcy and DCA ( Figure 3C). Additionally, the mRFP-GFP-LC3 adenovirus infection assay demonstrated a significant increase in both mRFP puncta and GFP puncta in macrophages treated with DCA ( Figure 3D). These results collectively suggest that the PDH activator promotes autophagy in Hcy-induced macrophages.

### PDH plays an essential role in maintaining macrophage autophagy in ApoE ^–/–^ mice

**Figure 4:**
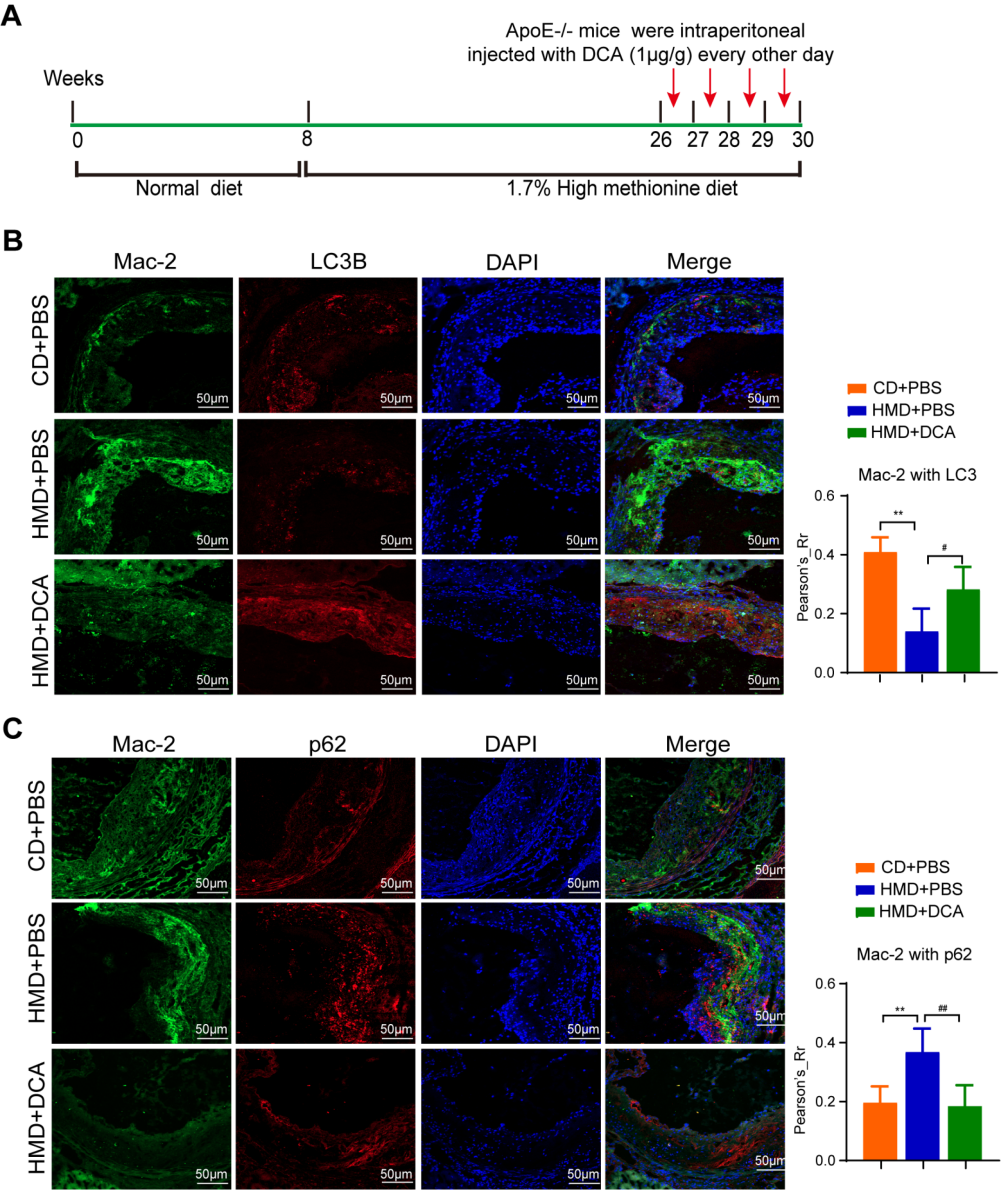
Figure 4 PDH activator facilitates macrophage autophagy in HMD-fed ApoE ^–/–^ mice (A) Model diagram of ApoE –/– mice that were intraperitoneally injected with DCA every other day and fed HMD. (B,C) Representative double immunofluorescence images showing the colocalization of LC3B (red) or p62 (red) with Mac-2 (green) in the atherosclerotic aortas of ApoE–/– mice (n = 6). Nuclei were stained with DAPI (blue) (scale bar: 50 μm). The quantification of the average intensity is shown in the right panel. Data are presented as the mean ± SD. **P < 0.01 CD + PBS group vs HMD + PBS group; #P < 0.05, ##P < 0.01 HMD + PBS group vs HMD + DCA group.

We also examined the effect of DCA on autophagy in HMD-fed ApoE ^–/–^ mice *in vitro*. Twenty-six-week-old ApoE ^–/–^ mice were injected with DCA via intraperitoneal injection every other day for 4 weeks ( Figure 4A). Double immunofluorescence staining revealed increased colocalization of LC3B (red, a marker for autophagy) and Mac-2 (green, a marker for macrophages) and decreased colocalization of p62 (red, a marker for autophagy) and Mac-2 in the aortic roots of HMD-fed ApoE ^–/–^ mice injected with DCA. Conversely, in HMD-fed ApoE ^–/–^ mice injected with PBS, LC3B expression was reduced, and p62 expression was elevated in the aortic root ( Figure 4B,C). These findings suggest that DCA can alleviate Hcy-induced inhibition of macrophage autophagy in ApoE ^–/–^ mice.

### PDH activator alleviates Hcy-induced autophagy via the AMPK/mTOR signaling pathway in macrophages

**Figure 5:**
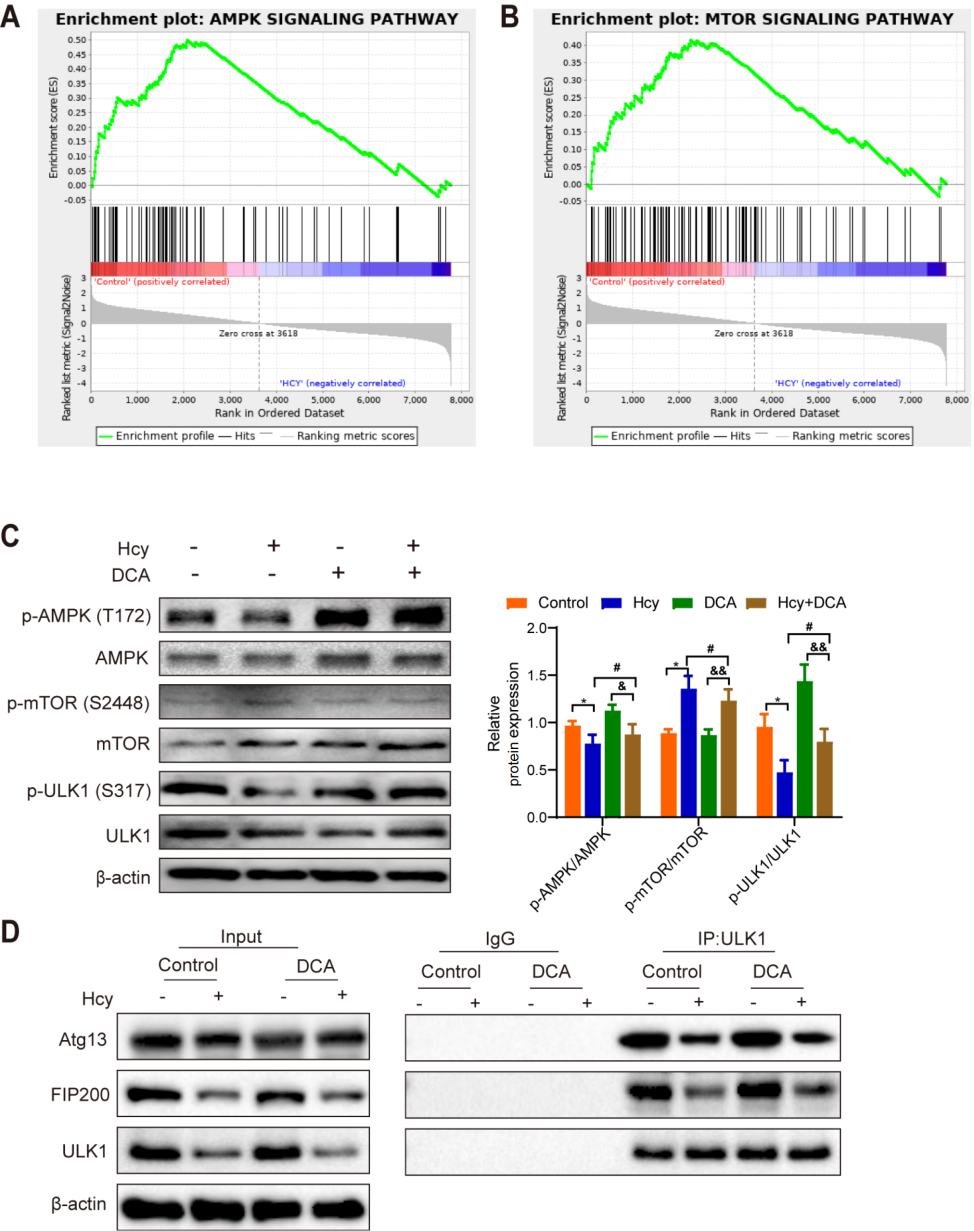
Figure 5 PDH participates in Hcy-mediated macrophage autophagy through the AMPK signaling pathway (A) GSEA of the AMPK signaling pathway in macrophages treated with or without Hcy. (B) GSEA of the mTOR signaling pathway in macrophages treated with or without Hcy. (C) The expressions of p-AMPK (T172), AMPK, p-mTOR (S2448), mTOR, p-ULK1 (S317), and ULK1 in macrophages treated with Hcy or DCA were detected by western blot analysis. (D) Co-IP assay showing the interaction between ULK1, Atg13 and FIP200 in macrophages treated with Hcy or DCA. Data are presented as the mean ± SD. *P < 0.05 Control group vs Hcy group; &P < 0.05, &&P < 0.01, DCA group vs Hcy+DCA group; #P < 0.05 Hcy group vs Hcy + DCA group.

The AMPK/mTOR pathway plays a pivotal role in the regulation of autophagy, particularly in cardiovascular diseases [ 26, 27]. To elucidate the precise mechanism by which Hcy inhibits autophagy, we investigated whether the differential expression of proteins was associated with the AMPK/mTOR signaling pathway, a crucial regulator of autophagy. Gene set enrichment analysis revealed significant enrichment of the AMPK and mTOR signaling pathways among the differentially expressed proteins (DEPs) ( Figure 5A,B). Subsequently, western blot analysis revealed reduced expressions of p-AMPK/AMPK and p-ULK1/ULK1 and increased expression of p-mTOR/mTOR in Hcy-treated macrophages. However, treatment with DCA significantly increased p-AMPK/AMPK and p-ULK1/ULK1 levels while decreasing p-mTOR/mTOR expression. Furthermore, DCA alleviated the suppressions of p-AMPK/AMPK and p-ULK1/ULK1 in Hcy-induced macrophages ( Figure 5C). ULK1, along with FIP200 and Atg13, forms the autophagy initiation complex. As shown in Figure 5D, co-immunoprecipitation assays revealed increased interactions between ULK1, FIP200, and Atg13 in DCA-treated macrophages, and these effects were reversed by Hcy treatment. Collectively, these findings suggest that Hcy inhibits macrophage autophagy by suppressing PDH activity through the AMPK/mTOR signaling pathway.

**Figure 6:**
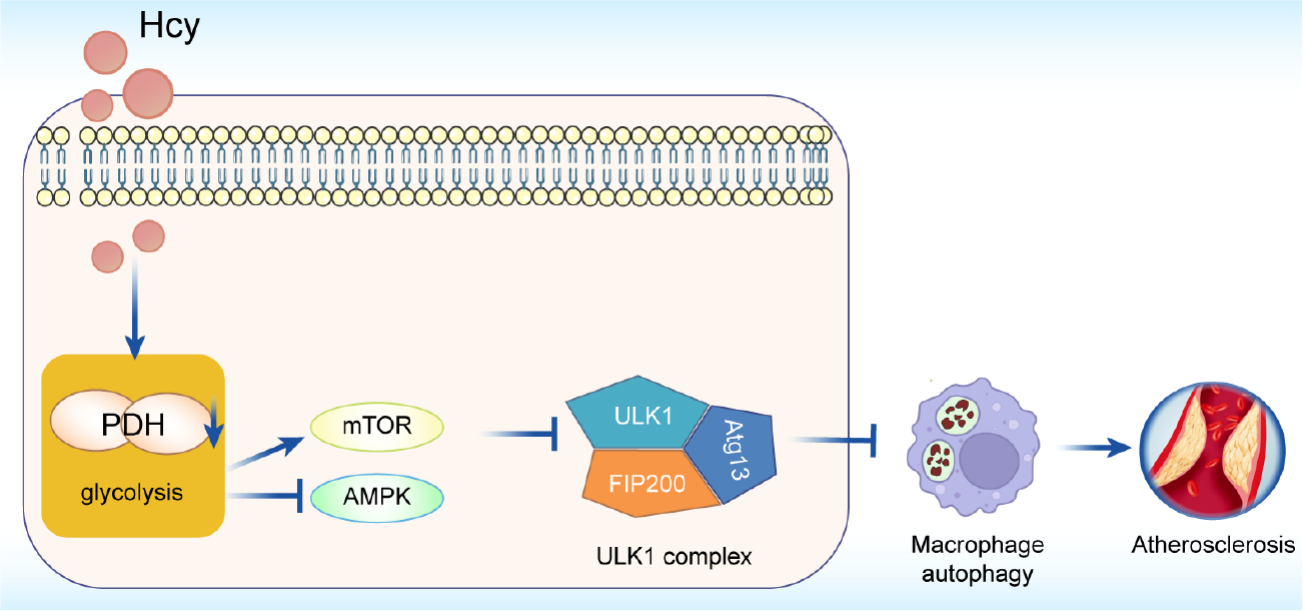
Figure 6 Schematic diagram illustrating the mechanism by which Hcy inhibits macrophage autophagy in atherosclerosis Hcy inhibits PDH activity and modulates the AMPK and mTOR signaling pathways to mediate the formation of the ULK1-FIP200-Atg13 complex, which suppresses macrophage autophagy during the progression of atherosclerosis.

## Discussion

Atherosclerosis, a chronic and multifactorial disease, remains a leading cause of morbidity and mortality worldwide. Substantial evidence indicates that macrophages play a critical role in its development and progression [5]. Homocysteine (Hcy), a sulfur-containing amino acid derived from methionine metabolism, has been identified as an independent risk factor for atherosclerosis [28]. However, the precise molecular mechanisms by which macrophages contribute to Hcy-induced atherosclerosis remain unclear. This study investigated the role of macrophages in the development of Hcy-induced atherosclerosis. Our findings revealed a reduction in PDH activity in Hcy-treated macrophages and revealed its role in regulating autophagy. These insights increase the understanding of atherosclerosis pathogenesis and suggest potential therapeutic targets for its treatment.

Autophagy is a conserved lysosomal pathway responsible for degrading cytoplasmic proteins and organelles, supporting the metabolic needs of cells and facilitating organelle renewal. p62 serves as a crucial link between autophagic substrates and LC3, directing autophagosomes to fuse with lysosomes. Impaired autophagic degradation leads to the accumulation of p62 [29]. Atherosclerosis begins with endothelial cell damage, with smooth muscle cells playing a key role in plaque stability, whereas macrophages are crucial in maintaining mature plaque stability and contributing to thrombosis formation [30]. Studies have shown that autophagy is involved in these atherosclerosis-related processes. Moderate autophagy can protect cells from apoptosis and necrosis by degrading damaged cellular structures and stabilizing plaques and inflammatory responses, thereby delaying or mitigating the progression of atherosclerosis [5]. However, the role of autophagy in macrophages in Hcy-induced atherosclerosis remains unclear. Our study demonstrated that Hcy stimulation significantly reduced macrophage autophagy in atherosclerosis, suggesting that modulating autophagy could be a potential therapeutic approach for treating or preventing Hcy-induced atherosclerosis. Western blot analysis further confirmed that Hcy inhibited macrophage autophagy in vitro. Notably, the expression of LC3BII and p62 was upregulated following treatment with bafilomycin A1, a compound that inhibits autophagosome-lysosome fusion, thus blocking autophagy and slowing the degradation of LC3BII and p62 [31]. These findings indicate that Hcy suppresses autophagy in macrophages, thereby potentially promoting atherosclerosis. Taken together, Hcy inhibits autophagy in macrophages, which may promote atherosclerosis.

PDH serves as the key enzyme linking glycolysis to the TCA cycle. Owing to its central role in metabolism, PDH has been recognized as a significant regulator of dynamic metabolic responses in various diseases [ 32, 33]. For example, Kaplon et al. demonstrated that reduced PDH activity through PDK1 overexpression in melanoma cells promotes tumor proliferation by enhancing glycolysis and reducing carboxylation [34]. Previous studies have also highlighted aerobic glycolysis as a critical metabolic pathway in endothelial and smooth muscle cells involved in atherosclerosis [ 35, 36]. Arvand et al. further reported that macrophage activation increases glycolysis through several activation steps [37]. Our findings align with these observations. Proteomic profiling revealed that Hcy enhances glycolysis, and we observed that the glucose content and pyruvic acid production were increased in macrophages treated with Hcy. Therefore, we hypothesized that glycolysis may be involved in the progression of atherosclerosis by regulating macrophage autophagy. In support of this hypothesis, we confirmed that the glycolysis inhibitor 2-DG reversed the inhibition of autophagy in Hcy-treated macrophages. Additionally, our results showed that Hcy inhibits PDH expression. PDH, an essential regulatory enzyme in glycolysis, acts as a bridge between glycolysis and the TCA cycle, which ensures the balance of cellular energy metabolism by regulating its activity, and we found that Hcy inhibited PDH activity in macrophages. Furthermore, si-PDH and the PDH inhibitor CPI-613 suppressed macrophage autophagy, whereas the PDH activator DCA alleviated Hcy-induced macrophage autophagy. Collectively, these results indicate that PDH plays an essential role in Hcy-induced macrophage autophagy.

The AMPK/mTOR pathway, which is activated by adenosine monophosphate, plays crucial roles in cell growth, proliferation, metabolism, and the regulation of autophagy. Increasing evidence has shown that the AMPK/mTOR signaling pathway is involved in cardiovascular diseases [ 38, 39]. Sun *et al*. [40] reported that impaired autophagy regulation through AMPK/mTOR inhibition is considered a potential cause of disease. Previous studies have demonstrated that AMPK/mTOR signaling not only regulates glucose, lipid, and protein metabolism across the body but also affects blood sugar and lipid levels in cardiovascular disease [41]. Additionally, Liu *et al* . [42] reported that AMPK/mTOR signaling regulates vascular endothelial and smooth muscle cell proliferation, migration, apoptosis, and autophagy while influencing macrophage function, which plays a central role in atherosclerosis. To explore the associations between specific proteins and the AMPK/mTOR signaling pathway in Hcy-treated macrophages, GSEA revealed a negative correlation between these genes and the AMPK/mTOR pathway. Additionally, western blot results further confirmed that Hcy inactivates the AMPK signaling pathway and activates the mTOR pathway. Autophagy is an evolutionarily conserved process, and its regulation involves various autophagy-related genes. ULK1, a key autophagy initiator, becomes enzymatically active and phosphorylates FIP200 and Atg13 to initiate autophagy [16]. Co-immunoprecipitation assays demonstrated that the expression of ULK1, FIP200, and Atg13 was reduced in Hcy-treated macrophages, whereas DCA treatment increased the expression of these proteins. These findings suggest that PDH is involved in Hcy-mediated macrophage autophagy through the AMPK/mTOR signaling pathway.

In summary, our findings reveal that Hcy suppresses autophagy by inhibiting the expression and activity of PDH. Additionally, we further demonstrated that a PDH activator regulates autophagy through the AMPK/mTOR signaling pathway in Hcy-induced macrophages ( Figure 6). These results highlight the need for further investigation into the potential mechanisms through which PDH regulates autophagy in macrophages exposed to Hcy.
